# Attachment of human periodontal ligament fibroblasts to root dentin conditioned with different endodontic irrigants: An experimental study

**DOI:** 10.34172/joddd.2022.002

**Published:** 2022-05-29

**Authors:** Elham Khoshbin, Rezvan Najafi, Maryam Farhadian, Maryam Khalili

**Affiliations:** ^1^Department of Endodontics, School of Dentistry, Hamadan University of Medical Sciences, Hamadan, Iran; ^2^Research Center for Molecular Medicine, Hamadan University of Medical Sciences, Hamadan, Iran; ^3^Department of Biostatistics, School of Public Health and Research Center for Health Sciences, Hamadan University of Medical Sciences, Hamadan, Iran

**Keywords:** Cell adhesion, Ethylenediaminetetraacetic acid, Periradicular surgery, Scanning electron microscopy

## Abstract

**Background.** Periradicular surgery is the last treatment option for teeth with persistent periradicular endodontic lesions. This study aimed to assess the adhesion of fibroblasts to root dentin conditioned with ethylenediaminetetraacetic acid (EDTA), MTAD, and QMix.

**Methods.** Twelve dentin discs were fabricated of 6 human single-rooted teeth. Fibroblasts were isolated from the periodontal ligament (PDL) of a premolar tooth. The teeth were healthy and freshly extracted from the socket. The samples were divided into four groups for surface conditioning with (I) EDTA, (II) MTAD, (III) QMix, and the control group. Fibroblasts were cultured on conditioned dentin discs at 37°C, 95% air, and 5% CO_2_ for 4 hours and then rinsed with PBS three times to eliminate unattached cells from the surface. The mean counts of attached cells were calculated using a Neubauer chamber. Also, the attachment of fibroblasts was evaluated using scanning electron microscopy (SEM).

**Results.** The mean counts of fibroblasts attached to root dentin in EDTA, QMix, MTAD, and control groups were 303±46, 243±41, 213±33, and 347±38, respectively. No significant difference was noted in the number of fibroblasts attached between MTAD, EDTA, and QMix and the control group (*P*>0.05). Under SEM, the fibroblasts were flat and spindle-shaped, with cytoplasmic processes covering the untreated dentin surface. In the experimental groups, the cells were rounder with fewer processes. All the three groups showed weaker adhesion to dentin compared to the control (untreated dentin) group.

**Conclusion.** Under the limitations of this study, it was concluded that treating the dentin surface with EDTA, MTAD, or QMIX might not be an effective way to improve the adhesion of human PDL fibroblasts.

## Introduction

 Dental pulp infection occurs due to caries, dental restorative treatments, or trauma and can lead to pulp necrosis, trigger the immune response, and eventually cause periapical lesions. The success of the management of these lesions with efficient endodontic treatment is as high as 90%. Thus, all inflammatory periapical lesions should be primarily managed with nonsurgical endodontic treatment.^1^

 Periapical surgery is the last resort as a treatment approach for teeth with persistent periapical lesions that do not resolve with conventional treatments. Periapical surgery is defined as cutting the part of the root end that has an un-debrided or unfilled (or both) root canal, followed by retrograde filling of the root canal of the remaining segment and achieving a hermetic root canal seal. Endodontic retreatment is the treatment of choice for such cases.^2^

 Following periapical surgery, efficient wound healing depends on re-establishing apical attachments and trabecular and cortical bone regeneration. Migration and adhesion of fibroblasts and deposition of cementum on the sectioned root surface are critical for proper healing of dentoalveolar tissues.^3^ Evidence shows that cell behavior is considerably affected by the surface properties such as surface roughness and topography.^4,5^ Following periradicular surgery, the adjacent cells are activated; they expand and migrate to the surgical site and produce growth factors and new matrix molecules.^6^

 In periodontal surgery, dentin demineralization leads to the formation of new connective tissue attachments. Thus, smear layer removal from the root end after sectioning is expected to enhance cementogenesis and promote periradicular wound healing. Root surface conditioning is performed for decontamination by removing the smear layer. In contrast, demineralization is performed to expose the collagen matrix of the respective surface to enhance the migration, proliferation, and attachment of fibroblasts and connective tissue fibers.^7^ This is done to expose the dentin collagen matrix while preserving the biologically active substances such as the growth factors in the dentin tissue.^8^

 To date, several materials have been proposed by researchers for smear layer removal, including ethylenediaminetetraacetic acid (EDTA), citric acid, polyacrylic acid, tannic acid, maleic acid, lactic acid, electrochemically activated water, sodium hypochlorite, RC-Prep, and phosphoric acid. However, none of these substances can serve as a gold standard for this purpose.^8-10^

 BioPure MTAD (Dentsply, Tulsa Dental, Tulsa, OK, USA) is a product commonly used to remove the smear layer and organic components from an infected root canal system. It is a mixture of a tetracycline isomer, citric acid, and detergent (Tween 80).^10^

 QMix (Dentsply, Tulsa Dental Specialties, Johnson City, TN) contains chlorhexidine, EDTA, and a detergent with a slightly alkaline pH.^11^

 There is insufficient information regarding the comparative effects of new products such as MTAD and QMix on the attachment of fibroblasts to conditioned root dentin. Thus, this study aimed to assess the attachment of human periodontal ligament (PDL) fibroblasts to root dentin conditioned with new endodontic irrigants.

## Methods

 This in vitro study was carried out in the Endodontics Department, School of Dentistry, Hamadan University of Medical Sciences, from February to August 2018.

###  Cell culture

 PDL fibroblasts were isolated from a 25-year-old healthy woman whose premolar tooth was extracted as part of orthodontic treatment. The tooth was extracted by a dental surgeon and stored in modified Eagle's medium (MEM), supplemented with 5% fetal bovine serum (FBS) (Gibco, Grand Island, NY, USA) and antibiotics, including penicillin and streptomycin (Sigma Chemical, St. Louis, Mo, USA).

 Periodontal tissue was scraped off the root using a sterile scalpel and rinsed with phosphate-buffered saline (PBS) twice. The scraped tissue was digested in a mixture of 0.05% trypsin and 0.02% EDTA in Hank’s balanced salt solution with decreased calcium and magnesium salts. Fibroblasts were cultured in sterile cell culture flasks (SPL Life, Science, Gyeonggi-do, South Korea), including Dulbecco’s modified Eagle’s medium, 10% FBS, 100 IU/mL penicillin, 100 ug/mL streptomycin, and 2 mM/mL glutamine in an incubator containing 5% CO_2_ and 95% air at 37°C. The culture medium was refreshed every 2-3 days during the cell culture process, and the cells were passaged after one week. After the 4th passage, the cells had a spindle shape and comprised a homogenous cell population.^12^ The cells were maintained in the culture medium under the mentioned conditions.

###  Flow cytometric analysis to confirm the expression of fibroblast surface markers 

 The expression of PDL fibroblast surface markers was evaluated using flow cytometry. The cells were transferred from PBS to flow cytometry color buffer. Five specific membrane markers were selected to confirm the type of cells, including the mesenchymal cell surface markers (CD90 and CD105), hematopoietic cell surface markers (CD34 and CD45), and STRO-1 as a mesenchymal stem cell marker. After trypsinization and rinsing with PBS, they were incubated with specific membrane marker antibodies at 37°C for 30 minutes and subjected to flow cytometric analysis. The nature and percentage of isolated cells were evaluated as such.^13^

###  Preparation of dentin slices

 Four single-rooted teeth and 12 dentin discs were used in this study. The extracted teeth eligible for inclusion had no cracks, fractures, caries, or resorptive defects at the apical third of the root. All the teeth were then rinsed with distilled water and stored in PBS at 37°C. First, the anatomical crown was cut at the cementoenamel junction with a diamond disc under water coolant. Next, two dentin blocks were sectioned from the apical third of the root of each tooth, measuring 3×4 mm with 1 mm of thickness, and UV-sterilized for 24 hours. Thus, 12 dentin slices were obtained as such, which were randomly divided into four groups for different surface treatments for 2 minutes: (I) 17% EDTA (MD-Cleaner, Meta Biomed, Chungju, Korea), (II) QMix (Dentsply, Tulsa Dental, Tulsa, OK, USA), (III) MTAD (Dentsply, Tulsa Dental, Tulsa, OK, USA), and (IV) control group with normal saline solution.

###  Evaluation of cell attachment

 After surface conditioning with the respective materials, dentin discs were washed with PBS and transferred to the culture medium to evaluate cell adhesion. To this end, 2500 fibroblasts were counted and transferred to each well containing a dentin slice. After four hours, dentin slices were transferred to a cell-free well. The dentin discs were rinsed with PBS at 37°C three times to eliminate unattached cells from the surface. The number of cells was counted using a Neubauer chamber (Assistant, Germany). The number of cells was counted in five squares of the slide, and the mean value was calculated. The mean value was subsequently multiplied by 10 000, and the number of cells per 1 mL of cell suspension was calculated. Since the cell suspension volume was 40 μL, the number of cells in the final volume was calculated by taking into account the coefficient of variation.^14^

###  Evaluation of cell attachment by scanning electron microscopy (SEM)

 One dentin disc along with the attached fibroblasts was randomly selected from each group for SEM evaluation. For this purpose, the selected dentin disc was rinsed with 37°C PBS twice and immersed in glutaraldehyde solution, dehydrated with ethanol, and dried. After sputter-coating with gold, the samples were evaluated under a microscope. The specimens were imaged by the BSE and SE detectors at different magnifications by the FEI ESEM Quanta 200 microscopy.

###  Statistical analysis

 The data were analyzed using SPSS 21 (SPSS Inc., IL, USA) via descriptive statistics and statistical tests, including the Kruskal-Wallis test. The level of significance was set at 0.05.

## Results

###  Confirming the expression of fibroblast surface markers

**Figure 1:**
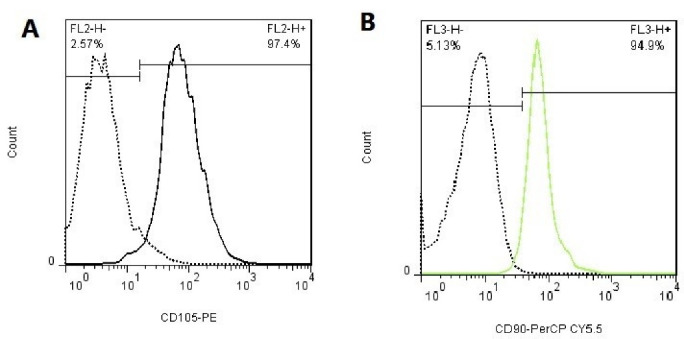
Expression of CD105 (A) and CD90 (B) markers.

 The expression of PDL fibroblast surface antigens was evaluated by flow cytometry. Evaluation of cell-specific surface markers revealed that the isolated cells resembled fibroblasts. Around 97.4% of these cells expressed CD105, and 94.9% expressed CD90 marker on their surface (Figures 1A and 1B).

**Figure 2:**
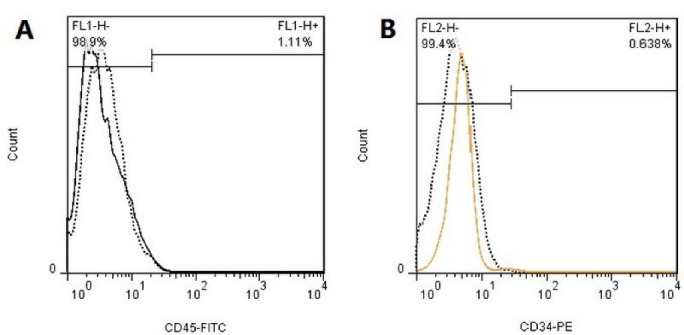
Expression of CD45 (A) and CD34 (B) markers.

**Figure 3:**
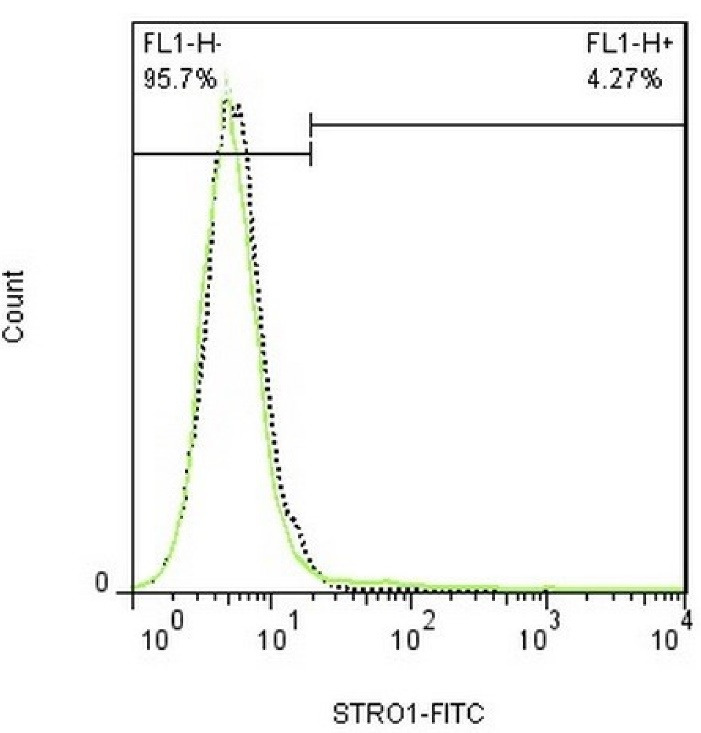
Expression of STRO1 marker.

 The cells were negative for the expression of hematopoietic cell markers such that approximately 1.11% expressed CD45 and 0.638% expressed CD34 marker on their surface (Figures 2A and 2B), indicating that they were mesenchymal cells. The cells were also negative for STRO-1 marker (4.27%), indicating that the cells were not stem cells but were fibroblasts (Figure 3).

###  Adhesion of fibroblasts to root dentin

 Assessment of the adhesion of fibroblasts to root dentin showed the highest mean adhesion in the control group (347 ± 38). Among the treated groups, the highest adhesion was noted in the EDTA group (303 ± 46), followed by the QMix group (243 ± 41). The MTAD group showed the lowest adhesion of cells (213 ± 33). The Kruskal-Wallis and chi-squared tests revealed no significant differences in fibroblast adhesion of EDTA, QMix, and MTAD groups compared with the control group (*P* = 0.092, Table 1).

**Table 1:** Mean number of fibroblasts adhering to root dentin conditioned with EDTA, MTAD, and QMix compared to the control group (n = 3)

	**Mean (SD)**	**Median**	***P*** ** value**
Control	347 ± 38	330	0.092
MTAD	213 ± 33	200	
EDTA	303 ± 46	320	
QMIX	243 ± 41	250	

*P* value was calculated by Kruskal-Wallis test.

###  Evaluation of fibroblast attachment to conditioned root dentin using SEM

**Figure 4:**
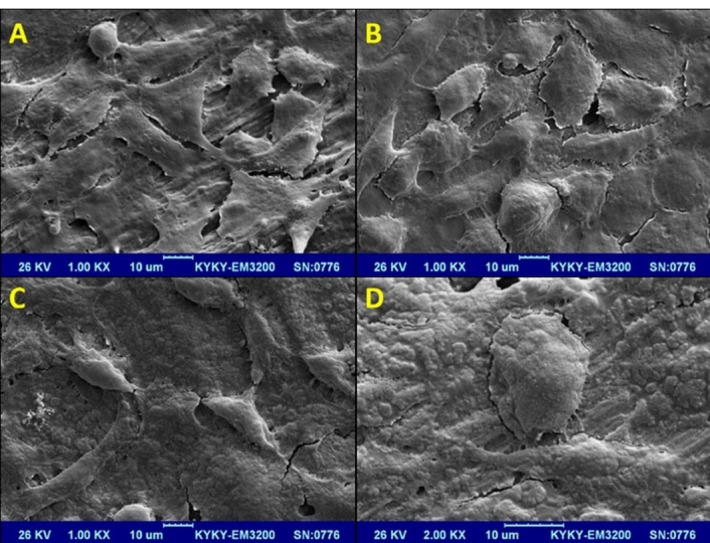
Attachment of fibroblasts to dentin: (A) control group, (B) conditioned with EDTA, (C) conditioned with QMix, and (D) conditioned with MTAD.

 In the control group (no dentin surface conditioning), fibroblasts were attached to the dentin surface, and a layer of fibroblast cells with their cytoplasmic processes was noted covering the dentin surface. Fibroblasts attached to dentin were flat and had a well-defined spindle shape (Figure 4A). The fibroblasts attached to dentin surface conditioned with EDTA had a slightly different morphology. It seemed that fibroblasts had lost their spindle shape to some extent (compared to the control group). However, they showed greater and more suitable attachment to dentin surface than the QMix and MTAD groups (Figure 4B). Fewer fibroblasts were noted attaching to dentin surfaces conditioned with QMix and MTAD than EDTA and control groups. Moreover, these fibroblasts showed a considerably modified morphology (Figures 4C and 4D).

## Discussion

 Periradicular surgery is the last treatment option for teeth with persistent periradicular lesions of endodontic origin, and fibroblast adhesion is an important step in re-establishing apical attachments and trabecular and cortical bone regeneration. This study evaluated the effect of EDTA, MTAD, and QMix on HPDL fibroblast attachment to the root dentin in vitro. The mean number of adhering fibroblasts was 347 ± 38 in the control (non-conditioned dentin) group. Among the conditioned dentin samples, the highest mean number of attached fibroblasts was noted in the EDTA group (303 ± 46), while minimum adhesion was noted in the MTAD group (213 ± 33). QMix had a mean adhesion of 243 ± 41.

 EDTA, QMix, and MTAD exhibited lower efficacy for fibroblast adhesion than the untreated dentin, although this difference did not reach statistical significance (*P* > 0.05).

 The untreated dentin, being a more efficient scaffold for cell attachment than conditioned dentin, was in contrast with some of the previous studies.^15-18^

 On the other hand, in a systematic review, Shewale et al,^19^ after reviewing 47 articles, concluded that root surface biomodifications are not beneficial in periodontal regeneration. Also, Prompreecha et al^20^ compared the effect of different dynamic irrigation methods with different solution regimens on apical papilla cells and showed that dynamic irrigation with normal saline solution results in significantly higher numbers of APCs than the same technique used with EDTA or chlorhexidine digluconate/EDTA solution regimen.

 In the present study, 17% EDTA was used for dentin surface demineralization. Babay’s^21^ study on the adhesion of fibroblasts following scaling and etching of root surfaces with periodontal disease showed that increasing the concentration of EDTA from 5% to 24% increases the adhesion of fibroblasts to root surfaces. The positive effect of 24% EDTA in the study by Silva et al^22^ on root dentin conditioning effects on periodontally affected teeth might also be due to the high concentration of the conditioning agent. In general, part of the difference in the results might be attributed to the different concentrations of EDTA used.

 In the present study, the adhesion of cells was evaluated after 4 hours. Rompen et al^23^noticed the adhesion and penetration of cytoplasmic processes of cells into dentinal tubules in all the conditioned and non-conditioned dentin samples in the first 2 hours, consistent with our findings. However, after 24 hours, the adhesion, proliferation, and biosynthetic activity of cells increased in the conditioned group. In the study by Babay,^15^thenumber of fibroblasts attaching to root surfaces conditioned by citric acid, tetracycline, and EDTA significantly increased after 72 hours. The duration of culture seems to have a significant effect on the results of different studies,^15,23^ as the surface treatment seems to have a bigger role in cell adhesion after the initial 4 hours.

 QMix is composed of chlorhexidine, EDTA, and surfactant. QMix is strongly capable of killing *Enterococcus faecalis* and bacterial plaque in planktonic form and biofilm. Its ability to eliminate the smear layer is comparable to that of EDTA.^24^ In the present study, the adhesion of cells in the QMix-conditioned group was less than the control group. Gianelli et al^25^ observed that chlorhexidine decreased the adhesion of fibroblasts to the fibronectin network and prevented the adhesion of cells to root dentin. Considering the optimal substantivity of chlorhexidine on dentin surfaces,^26^ lower adhesion of cells in the group conditioned with QMix can be attributed to the presence of chlorhexidine in its composition. However, no study has directly evaluated the effect of QMix as a conditioning agent on the adhesion of fibroblasts following periapical surgery, and further studies are required to assess its effect on cell adhesion.

 MTAD was introduced by Beltz et al, in 2003. It is used to remove the smear layer and organic contents of infected root canals. It contains tetracycline isomer, citric acid as a demineralizing agent, and a detergent (Tween-80). MTAD effectively eliminates the smear layer, can dissolve the pulp tissue, has antibacterial properties, and is effective against *E. faecalis*.^27,28^

 Our results showed that the number of cells attaching to dentin conditioned with MTAD was less than the control group. Ghandi et al^29^ evaluated the effect of MTAD on the adhesion of fibroblasts to root surfaces and showed that the mean number of fibroblasts attached to roots conditioned with MTAD was less than the control group; however, this difference was not statistically significant. Some of the constituents of MTAD have been used as conditioning agents. Babay^15^observed improved cell adhesion following surface conditioning with EDTA and tetracycline. Chandra et al^30^demonstrated the positive effects of root conditioning with citric acid and EDTA; however, tetracycline did not yield positive results. Wikesjö et al^31^ showed that the healing events were the same around dentin surfaces conditioned with citric acid and untreated dentin surfaces. Fewer cell attachment after treating the surface with MTAD might indicate that the presence of the smear layer on root surfaces is not a barrier against the attachment of fibroblasts,^29^ consistent with our findings, and in agreement with those of Delazari et al^32^ and Al-Nazhan.^12^

 In the process of cell attachment, the shape of cells transforms from a round shape with microvilli to a flat shape with more stable phyllopodia over time. Such transformations occur in different phases of a continuous process. The duration of these phases and their degree of superimposition might vary among different cell lines on different substrates.^33^ The quality of cell adhesion is determined by several characteristics in the SEM view. Varghese and Ballal^34^ consider a cell attached when the cell’s nucleus is in touch with the dentin surface or when the cell is located in the intercellular matrix attached to the dentin surface. In various other studies, the flat shape of cells and the increased number of cytoplasmic processes define cell adhesion to the surface.^6^ In the present study, such evidence was more commonly observed in the control and EDTA groups compared to QMix and MTAD groups. Cells adhering to the surfaces were flat and spindle-shaped, with cytoplasmic processes covering the dentin surface as a monolayer. Rajaraman et al^33^also observed that in the process of fibroblast adhesion to dentin, cells transformed from a round form with microvilli to flat cells with more stable phyllopodia; the attachments became more stable following flattening of the cells and the formation of cytoplasmic processes. Al-Nazhan^12^ evaluated the adhesion of human PDL fibroblasts to non-demineralized dentin in vitro and noticed that fibroblasts had optimal adhesion to non-demineralized dentin even in the presence of the smear layer. This finding highlighted the biocompatibility of dentin surfaces despite the presence of the smear layer.

 Some limitations should be noted in this in vitro study. The effect of the conditioning agents on the HPDL fibroblast adhesion was assessed at 4 hours, which might be considered a short time. Moreover, only a certain concentration of EDTA (17%) was used in our study. It is suggested that studies with different concentrations of the conditioning agents, after several durations of cell culturing on the root dentin, should be undertaken to specify the role of the agents in cell adhesion. In the current study, the endodontic irrigants’ effect on root dentin was tested. It would be interesting to compare it with other methods of root dentin conditioning, such as antimicrobial photodynamic therapy or laser therapy.

## Conclusion

 Considering the limitations of the study, current results suggest that conditioning the dentin with EDTA, MTAD, and QMix during the root-end surgery, might not improve the adhesion of HPDL fibroblast cells to the root surface. Considering the significant advantages of using these agents in the process, such as the triggering effect of EDTA on growth factors release or the efficacious antibacterial features of MTAD and QMix, further studies, especially in vivo studies, should be carried out before judging the application of these agents in the clinic.

## Acknowledgments

 This investigation is a part of the thesis of Maryam Khalili, approved and financially supported by the Vice-chancellor of Research and Technology of Hamedan University of Medical Sciences, Hamedan, Iran.

## Authors’ Contributions

 MK and EK performed all the experiments and analysis and also contributed to writing the manuscript. RNand MFcontributed as a supervisor and revised the manuscript. EK carried out critical revision.

## Funding

 This article was financially supported by the Vice-Chancler of Research and Technology of Hamedan University of Medical Sciences, Hamedan, Iran.

## Competing Interests

 The authors declare that they have no known competing financial interests or personal relationships that could have influenced the work reported in this paper.

## Ethics Approval

 The study was approved by the Ethics Committee of Hamadan University of Medical Sciences (IR.UMSHA.REC.1397.126).
